# Involvement of bone in disseminated paracoccidioidomycosis

**DOI:** 10.1590/0037-8682-0620-2020

**Published:** 2021-03-08

**Authors:** Philippe Alcântara Gonçalves Martins, Rosana Souza Rodrigues, Miriam Menna Barreto

**Affiliations:** 1 Universidade Federal do Rio de Janeiro, Departamento de Radiologia, Rio de Janeiro, RJ, Brasil.; 2 Instituto D’Or de Pesquisa e Ensino, Departamento de Radiologia, Rio de Janeiro, RJ, Brasil.

An 18-year-old male patient previously healthy and immunocompetent presented with a 6-months history of cough, sputum production, malaise, and weakness. The patient had fever, diffuse lymph node enlargement, multiple cutaneous lesions, bone pain, and a purulent collection of on the thoracic wall adjacent to the left clavicle. He reported a weight loss of about 10 kg in just one month.

**FIGURE 1:**
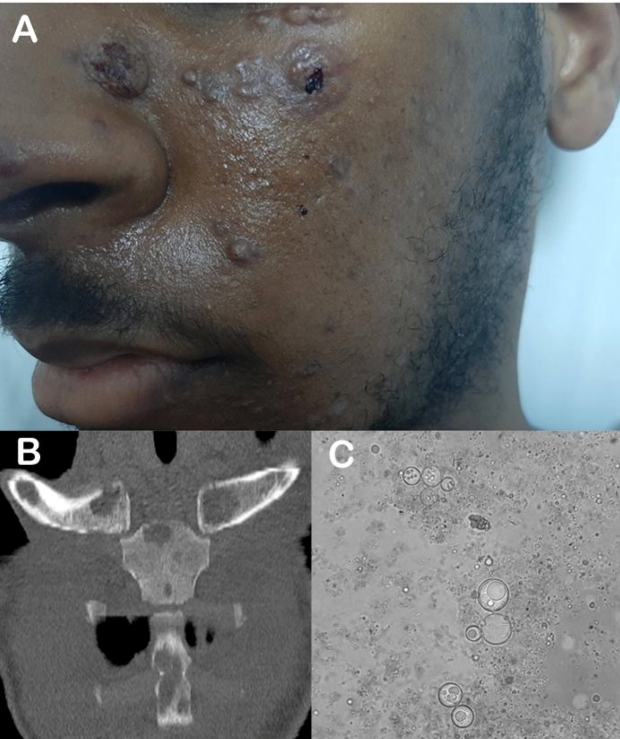
(A): Patient with subacute disseminated form of paracoccidioidomycosis exhibiting multiple umbilicated skin-colored papules on the face. (B): Coronal multiplanar reformatted computed tomography of the anterior chest wall demonstrating the presence of multiple osteolytic lesions with cortical rupture of the sternal body, manubrium, and both clavicles. (C): A photomicrograph showing the thick birefringent cell wall of the fungus and the typical pattern of multiple budding around the mother cell, which is a characteristic feature of *Paracoccidioides brasiliensis* infection*.*

Physical examination revealed the presence of umbilicated skin-colored papules on the face (Figure 1A), neck, and upper limbs. He tested negative in the sputum test for pulmonary tuberculosis and serological tests for human immunodeficiency virus (HIV).

Contrast-enhanced computed tomography (CT) of the chest revealed lymph node enlargement and the presence of multiple osteolytic lesions on the sternum and both clavicles (Figure 1B). The pulmonary parenchyma was normal. It was diagnosed as disseminated paracoccidioidomycosis (PCM) based on the presence of osteolytic lesions, diffuse lymph node enlargement, and cutaneous lesions in an epidemiological setting.

However, the diagnosis of PCM was confirmed upon identification of *P. brasiliensis* in the sample obtained from the chest aspirate (Figure 1C). The patient was diagnosed with a severe juvenile subacute form of PCM. He was administered with liposomal amphotericin B and showed substantial clinical improvement, with weight gain and improvement in the cutaneous and osseous lesions.

Involvement of bone in PCM is uncommon and results primarily from hematogenous dissemination. The most commonly affected sites are the chest bones (i.e., the ribs, sternum, clavicle, and scapula), as observed in our case. Bone lesions in PCM are usually osteolytic, with no sclerotic rim or periosteal reaction^1^ ^-^ ^3^. Although nonspecific, the imaging results of osseous lesions suggest PCM infection in appropriate clinical and epidemiological settings.
